# Situational context of home-based sexual education in urban slums of Ibadan, Nigeria–evidence from a qualitative study

**DOI:** 10.1371/journal.pone.0304200

**Published:** 2024-06-17

**Authors:** Taofeek Kolawole Aliyu

**Affiliations:** 1 Department of Sociology and Anthropology, Obafemi Awolowo University, Ile-Ife, Nigeria; 2 Department of Sociology, Centre for Demographic and Ageing Research (CEDAR), Umeå University, Umeå, Sweden; Indiana University School of Medicine, UNITED STATES

## Abstract

This study explores the influence of situational context on parent-adolescent communication about sexual and reproductive health (SRH) issues in the urban slums of Ibadan, Nigeria. A qualitative exploratory study was conducted in the Southeast and Northeast LGAs of Ibadan. Eight (8) vignette-based focus group discussions (FGDs) with parents and adolescents of both sexes were conducted in addition to four (4) key informant interviews (KIIs) with community and women’s leaders. Interviews were tape-recorded, transcribed, and translated into English. Thematic analysis was adopted using ATLAS Ti 9 software. Findings portray SRH meanings and experiences, intergenerational cultural norms, and expectations for SRH, gender double standards in SRH discussion, streetwise SRH knowledge, and social media exposure as contexts that interfere with parent-adolescent communication on SRH issues. The findings show that despite understanding the need for SRH discussion, parents and adolescents lack effective communication on SRH issues due to the interference of unfiltered streetwise SRH knowledge and social media exposure. Also, SRH conversation between parents and adolescents promotes gender inequalities as different information is passed to adolescent girls and boys. Interventions that take into account situational occurrences must be geared towards enabling parents to give their adolescents early exposure to relevant, context-specific SRH knowledge.

## Introduction

Context-based sexual education, which often begins at home, has the potential to influence adolescents’ sexual behaviour as a critical component of socialisation. This approach recognises that sexuality is a complex and multifaceted aspect of human life and considers the individual, social, and cultural contexts of socialisation. As a component of sexual socialisation, parent-adolescent communication about sexual issues gives parents the opportunity to mirror the life circumstances in their conversation and connect adolescents to the sexual values and ideals of their environment. Studies affirmed that parents do not just passively act out their internalised values and ideals about sexual issues; they also proactively employ their understanding of SRH issues to shape their adolescents’ behaviours [1, 2]. By engaging in open and honest communication about SRH issues, parents can provide their adolescents with accurate information, promote healthy attitudes towards sex [3–5], and help to prevent negative consequences such as unintended pregnancy, STIs, and HIV/AIDS [6–9].

Despite the widely acknowledged importance of parent-adolescent SRH communication in ensuring healthy sexual outcomes in adolescents, the socio-cultural norms, conservative traditions, and environment that promote it are nevertheless sensitive, contentious, and blurred by complex moral barriers and intergenerational boundaries. This has resulted in conflicting ideologies about what and how to communicate regarding adolescent sexual behaviours, including sexual initiation, exploration, and/or abstinence and its implications for adolescent SRH outcomes [10, 11]. Existing literature contends that prevailing social norms and ideologies prevent parents from effectively discussing SRH issues with their adolescents [12–16]. Also, parents’ socio-cultural and religious beliefs that adolescents are too young to have conversations on sensitive matters like SRH issues and the uncomfortable atmosphere that results from such dialogue are additional barriers to SRH communication [9, 17, 18]. Studies have highlighted the detrimental effects of a knowledge gap in sexual education as a factor in the rise of risky adolescent sexual behaviours and adverse SRH outcomes such as unplanned pregnancy, abortion, STDs/STIs, and HIV/AIDS [19–22]. They argued that adolescents will make informed and healthy decisions if they are equipped with the SRH knowledge and values necessary to realise their rights to sexual and reproductive health and well-being.

There is a growing body of literature on the protective role of parents in sexual socialisation and in promoting healthy sexual development in adolescents, including delayed sexual debut, fewer sexual partners, preventing unintended pregnancy, using contraception more consistently, and making informed decisions about SRH. In the United States, a large body of literature has shown the positive effect of parent-adolescent communication on sexual issues [23–26]. Evidence from Sub-Saharan Africa (SSA) suggests that parent-child dialogue on SRH issues is becoming more common [8, 14, 27–30]. However, the conversation continues to focus more and more on abstinence, sometimes laced with cultural and religious biases [18, 19, 31], without reflecting the contemporary reality of adolescents about sexual issues. Additionally, a number of factors or barriers that prevent effective parent-child discussions about SRH have been examined in research conducted in Ghana [27, 32–34], Ethiopia [15, 19, 35, 36], Kenya [29], South Africa [37, 38], and Nigeria [8, 28, 30]. Even though these studies have demonstrated some effects, there is little empirical evidence about the situational context of parent-adolescent communication regarding SRH issues in urban slums. Existing studies have confirmed that adolescents in slum face a higher vulnerability to SRH issues, such as early sexual initiation, unprotected sexual intercourse, multiple sexual partnerships, transactional sex, sexual exploitation, and abuse. These issues are exacerbated by prevalent conditions in urban slums, including poverty, limited education, unemployment, ineffective parental communication and support, and detrimental societal pressure [39–42]. The current study provides new evidence and contributes to the body of literature by investigating the situational context that interferes with and undermines parents’ ability to effectively communicate about SRH issues with their adolescents. The study complements the literature on parent-adolescent communication regarding SRH in two ways. One, it extends the body of literature by providing insight into how parents and adolescents understand and experience SRH in the context of intergenerational cultural differences and social media proliferation, which shape the discourse and explain why many parents and adolescents find it challenging to openly discuss sexual-related topics. In this regard, this study draws on the social constructionist perspective, which emphasises how society creates realities for individuals and the role of people as context-sensitive actors. Second, through a vignette-based qualitative approach, this study draws data from economically deprived settings that are known to have poor outcomes for adolescents’ sexual and reproductive health rights in addition to limited access to SRH services. This is critical in the light of the current context, in which most cities are experiencing rapid urbanisation along with the growth of informal settlements. With few exceptions [18, 28, 29, 38], the literature on parent-child communication on sexual issues has either treated urban settings as being homogenous [36, 43], relied on data from rural areas [6, 44], or ignored economically deprived settings when investigating parent-adolescent communication about SRH issues. In contrast, exploring the interplay of informal settings and situational context on sexual communication is a step forward in parent-adolescent communication about sexual issues. The slum setting is a theoretically intriguing case to explore due to its typical characteristics of high population concentrations, poor living conditions, restricted access to healthcare facilities, and insufficient social services. Again, the lack of privacy is as a result of overcrowding, where families have limited space to hold conversations on SRH without their neighbours eavesdropping. Accordingly, this study explores the situational context of parent-adolescent communication on SRH issues in urban slums in Southern Nigeria. Understanding the situational context of home-based sexuality communication between parents and adolescents is important both from a research- and policy-related perspective.

## Theoretical framework

The social constructivism theory of Berger and Luckmann’s (1991), which contends that knowledge is produced through social interactions and cultural contexts rather than being simply conveyed from an objective reality to a person, served as the framework for this study. Building on the substructure of [45] framework, [46] limit their focus on the actor-in-social-contexts, explaining how individual behaviour or action is a product of learning from social interaction. That is, having a place in the society and constructing individual identity therein, originate from interacting with and learning from the significant others who interpret the constructed reality and render it meaningful to an individual in the society [46]. Applied to the context of parent-adolescent communication on sexual issues in slums, social constructivism helps explain how communication about SRH issues is influenced by the prevailing socio-cultural norms and the situation of the community. In slum communities, the situations that prompt sexual issues and the socio-cultural norms around them may differ from those in other contexts and may be shaped by factors such as poverty, limited access to healthcare, and traditional gender roles. The interplay of these socio-cultural ethos can influence how parents and adolescents communicate about sexual issues, as they may impact what is considered appropriate to discuss openly and what topics are considered taboo. For example, if the community has a strong cultural norm of silence around sexual issues, parents may be hesitant to discuss these topics with their adolescent children or may only do so in private settings (often not available). It can be challenging to maintain privacy in slum areas when resources and infrastructure may be limited, and most homes are overcrowded. Social constructivism also suggests that communication is a dynamic process that involves negotiation and collaboration between individuals. In the context of parent-adolescent communication on sexual issues, this means that both parents and adolescents may bring their own perspectives and beliefs to the conversation, and that communication is likely to be most effective when both parties are able to listen to and understand each other’s points of view. In contrast, if both the adolescents and parents share different meanings on what constitutes the appropriate information on SRH issues, they are likely to develop a negative attitude towards communication. Additionally, the intergenerational variations in sexual issue experiences and earlier communication experiences may influence the current conversation between parents and adolescents. The assumption is that both parents and adolescents have a different worldview and the meaning they ascribe to their respective worlds must be understood for proper communication to take place. Given that adolescents are now living in a world entirely different from that of their parents, characterised by technological advancement, they are likely to rely on sources like social media for information on SRH issues, particularly when social media provides a veritable platform to express their realities freely.

## Methodology

### Study settings

This paper is based on a qualitative study conducted in Ibadan Northeast and Southeast Local Government Areas (LGAs) from 26^th^ April to 2^nd^ June 2018 among parents, adolescents (10–19 years), community leaders, and women opinion leaders. Ibadan is the capital of Oyo State in Southwest Nigeria and a metropolis of more than 3 million people. The city is divided into five (5) Local Government Areas (LGAs), two of which contain the highest concentration of slums, namely, Ibadan Northeast and Southeast. The two LGAs have comparable cultural practises and beliefs. As submitted by [47] and [48], which also resonate with the reality of the study participants, the conditions of slum dwellers in Ibadan are exceedingly poor, often with limited access to basic services including basic healthcare services, portable water, and proper waste management and disposal. A pre-field visit showed that the study area is characterised by poor living conditions, a high concentration of dilapidated buildings, bad roads, lack of toilet and bathroom facilities, open defecation, improper housing structures, inadequate infrastructure, overcrowding, improper waste, street trading and hawking to mention a few. Also, residents of the study area have access to low-cost devices and the internet through network providers like MTN, Globacom, Etisalat, and Airtel. Hence, it is anticipated that the dynamics of parent-adolescent communication about SRH issues will be influenced by their social context and prevailing cultural norms and values pertinent to the survival of the residents.

### Research design and sampling procedure

This study embraced an exploratory qualitative design. Participants were selected using a purposive sampling procedure. The initial stage involved the identification of the most populous slums with inhabitants in the two LGAs using the enumeration area (EA) maps from the National Population Commission (NPC). This was made possible with the assistance of two employees of the NPC. Subsequently, two communities (one from each LGA)–Aremo from Ibadan Northeast and Beere from Ibadan Southeast were selected. The inclusion criteria for selecting the participants include gender, age, ethnicity (Yoruba), and location. Participants from varied socioeconomic backgrounds and the three most prevalent religions in the area were actively sought out to ensure relative representation. Four seasoned research assistants (male and female), assisted in the data collection.

### Data collection

The Institutional Review Committee of the author’s institution granted ethical approval for this study, which was conducted between April and June 2018. Data were gathered using a vignette-based focus group discussions (FGDs) and key informant interviews (KIIs). A pre-test of the FGDs and KIIs guides was conducted in Ile-Ife, Osun State, with selected parent-adolescent pairs. This preliminary testing was instrumental in refining and finalising the instruments before their deployment in the main study. In the two selected communities, there were a total of 8 FGDs (4 each) with parents and adolescents (of both sexes, aged 15–19), as well as 4 KIIs (2 each) with community leaders and women opinion leaders (see Table 1). The study ensured that an equal number of FGDs and KIIs were conducted in both communities. Each FGD session comprised a minimum of 8 and a maximum of 12 participants for both parents and adolescents. The FGDs had 73 participants because a few participants dropped out in the middle of the discussion. A gatekeeper and a community leader assisted in recruiting the participants, who had to be parents and adolescents who had lived in the community for at least two years. The groups were organised based on gender for both parents and adolescents to ensure homogeneity and encourage active participation in the discussions. As a result, two FGD sessions with parents and adolescents, one for males and one for females were held. A vignette guide created by the researchers was used by trained Research Assistants (RAs) with a minimum of a Bachelor of Science degree to moderate FGD sessions, which were audio-taped. The data were collected by four (4) RAs under the supervision of the researcher and a female colleague. One designated gatekeeper from each community assisted with community entry and participants recruitment. The gate keepers were identified during a pre-field visit and were contacted on phone subsequently to schedule appointments with recruited participants. The gatekeeper in each community identified the men and women who fall within the inclusion criteria. The RAs introduced themselves before the interviews began, and the participants did the same to establish a rapport and help them feel at ease after which the purpose of the study was introduced. Each FGD lasted almost an hour. A conducive environment and refreshments for the participants were provided to guarantee successful FGD sessions. Each FGD was facilitated by one moderator and one notetaker.

**Table 1:** Summary of the FGDs and KIIs.

Participants	Beere		Aremo	
Parents	FGD	KII	FGD	KII
Fathers	1		1	
Mothers	1		1	
**Comm. Leader**		1		1
**Women Leader**		1		1
**Adolescents**				
Boys	1		1	
Girls	1		1	
**Total**	**4**	**2**	**4**	**2**

The KIIs involved sourcing for community leaders and women leaders to have a balance of gender in their opinion about the situational context of parent-adolescent SRH communication. One community leader and one women leader were interviewed in each community making a total of 4 KIIs. A KII guide was used for community leaders and women leaders which involved an audio recording of the interview sessions as well as note-taking. The interviews were held in the language (local language) understood and preferred by the key informants. The interviews took place in a conducive atmosphere as chosen by the informants. The informants were provided with refreshments after their sessions.

A pre-test of the vignette-based FGD guide and KII guide was conducted in Ile-Ife with male and female participants who share similar characteristics with the study participants. Observations and corrections noted during the pre-test were used to correct and restructure the final FGD and KII guides.

### Vignette-based FGD guide

The qualitative data were captured using a structured vignette to prompt engagements with the story in a focus group discussion which was developed based on specific themes. The choice of this method was premised on the fact that participants might give a normative answer instead of an honest answer especially about sensitive issues such as issues of SRH. Although, the study’s aim is not to question people about their sexuality, some questions might be interpreted as such. Thus, the rationale for opting for a vignette-based FGD was to prevent participants from feeling that there was a "right" or "wrong" answer and to allow them to talk freely about sexual issues. It also helped to probe the dynamics of the issue further, thereby gaining a more detailed insight into issues under investigation among slum dwellers. Discussants were encouraged to discuss by telling stories about SRH issues between Adio (male) and Abeni (female). Questions were asked about Adio and Abeni in relation to adolescents’ sexual behaviours and their perception of the story.

### Data analysis

**Fig 1:**
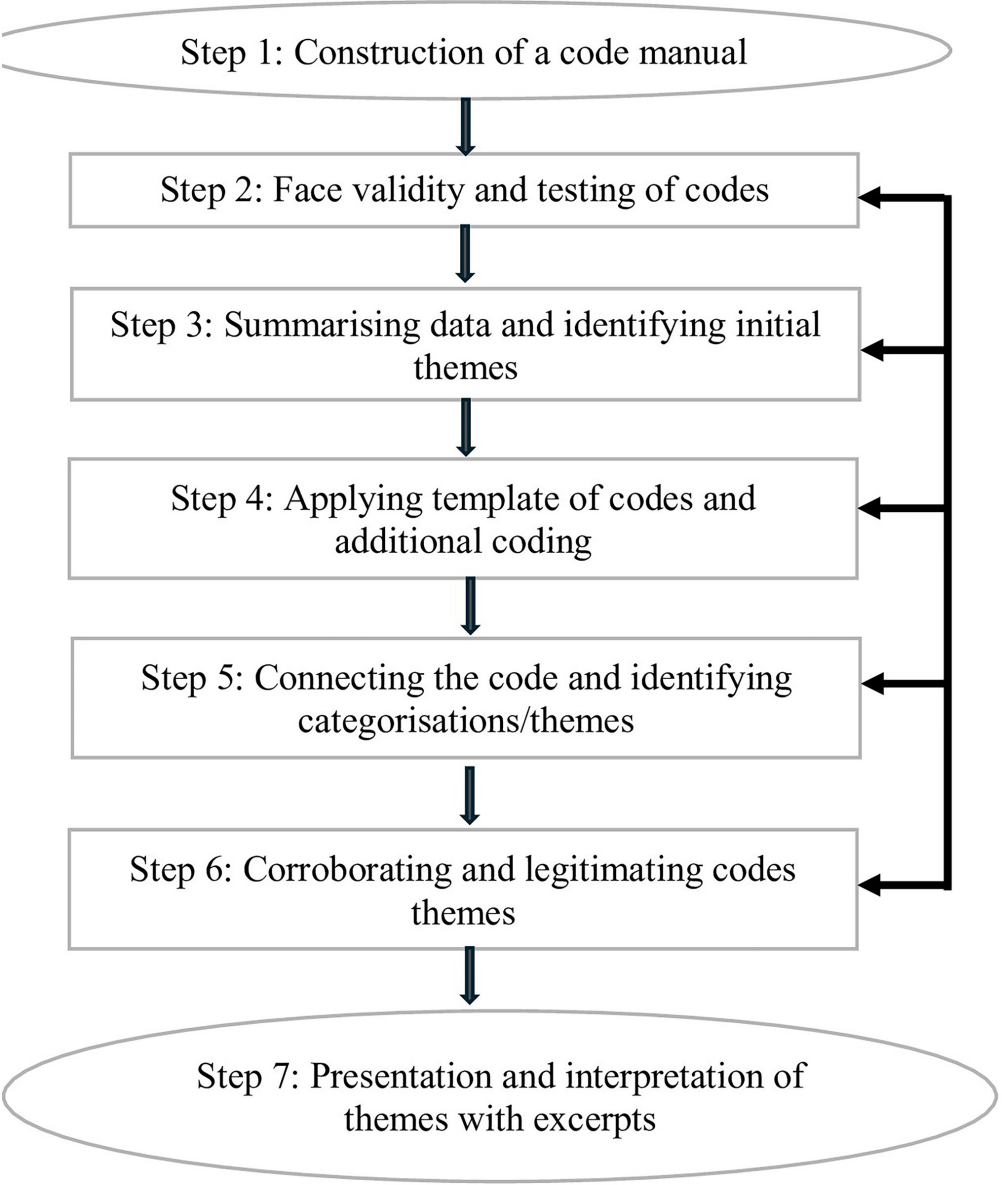
Diagrammatic representation of data analysis procedures and steps [49, 50].

All discussions and interviews were transcribed verbatim, and those that were held in the native tongue were transcribed verbatim before being translated into English. The analysis progressed in accordance with a seven-step approach recommended in the existing literature [49, 50] as shown in Fig 1. The steps entail guaranteeing the accuracy of every interview that was transcribed and translated. Then, an expert in both languages reviewed the transcriptions and translations to make sure the participants’ viewpoints were properly and accurately represented. Thereafter, familiarisation with the data was undertaken as the transcripts were read multiple times in search of recurring ideas and differences in thoughts to ensure that codes were developed in line with the data. These steps culminated into the creation of a codebook. The created codebook was imported to ATLAS ti. software. The data coding process progressed by applying codes from the codebook to relevant segments of the transcripts. It was guided, though not confined, by these preliminary codes as new codes emerged during this process. In addition, memos were also utilised to record potential iterations, thoughts, and queries to help describe and explain the meaning of codes. To ensure intercoder reliability, some portions of the transcripts were shared with an expert in qualitative research. The shared transcripts and developed codes revealed an 80% agreement, and ambiguous codes were subsequently discussed and resolved using the focus coding strategy advocated by [51]. The coded data were merged and organised into broad categories and sub-categories. The main categories were then clarified by going over all the data gathered from the FGDs and KIIs. Similarities and differences between categories/themes emerged at this stage, indicating areas of consensus in response to the research questions. As a result, five broad categories that emerged from the data include: SRH meanings and experiences; intergenerational cultural norms and expectations of SRH; gender double standards in SRH; streetwise SRH knowledge; and social media exposure to SRH. This process ends ended with presentation and interpretation of these categorisation/themes.

### Ethical considerations

The Obafemi Awolowo University, Ile-Ife Institutional Review Board approved this study (HREC No.: IPH/OAU/12/983). Participants were assured of voluntary participation and were given the option to withdraw at any point they deemed fit. Pseudo-names were assigned to FGD participants to ensure their anonymity. Verbal and written Informed consents were obtained from participants 18 years of age and older, while assents were obtained from adolescents younger than 18 years of age. Consent from parents or guardians was obtained before interviewing adolescents. To ensure the confidentiality of the data, the files (audio and transcripts) were saved on a password-protected computer, and only the researchers had access to them.

### Rigor of the study

The study’s credibility was ensured through the application of purposive sampling, which involved selecting participants with direct experiences of the issues under investigation and a willingness to openly share their experiences. As such, trustworthiness was established through prolonged engagement with participants to ensure accurate data collection in both KIIs and FGDs. Probing techniques were employed to enhance the depth and robustness of the research findings. Member-checking was implemented during interviews to validate emerging themes and ensure the consistency and authenticity of the data collected. Comprehensive descriptions of participants’ characteristics and context were provided to facilitate transferability for researchers interested in replicating this study. Dependability was assured by involving an independent data analyst who analysed and verified the findings.

## Results

### Socio-demographic profiles of participants

#### Focus group discussants

In the homogeneous vignette-based FGDs, 33 parents (fathers and mothers) participated, with seventeen from Ibadan Northeast LG and sixteen from Ibadan Southeast LG. Twenty-three of the participants were between the ages of 40 and 49 years. A very small number of the participants were either divorced (8) or widowed (3), whereas 22 of them were married. All the parents (33) were educated, and their level of literacy ranged from elementary to secondary levels. Most of them (20) were parents to between 4 and 6 children, while others had less than 4 children. Out of the parents, 19 are Muslims and 14 are Christians. About 22 participants reported that business or commerce was their main source of income.

On the other hand, there were 40 adolescent participants in this study, with 19 from Ibadan Northeast and 21 from Ibadan Southeast. The 40 adolescents were all in their late adolescence (15–19 years). They were fairly gender-balanced, with 19 boys and 21 girls. Among them, 11 acknowledged dating, and the majority (29) were single. Thirty (30) adolescents claimed to have secondary education, while 10 had completed elementary school. Among the adolescents, 23 are Christians and the other 17 are Muslims.

#### Key informant interviewees

With consideration for their locations, gender, and social position in the community, four key informants were evenly represented. While two of the interviewees were married, the other two were either widows or widowers. Three key informants had secondary education, and one had primary education. Three of the informants were Muslims, while the last person is a Christian. Three (3) of the participants were business owners, and just one participant was a farmer.

### Situational context of SRH communication

Understanding the situational context, that is, the specific circumstances, settings, or conditions in which discussions, conversations, or exchanges about SRH take place, is crucial for effective SRH communication between parents and adolescents. The findings revolved around five interconnected themes. The first theme provides insights into the interpretative constructions of meanings and experiences surrounding SRH. The emphasis here was on how both parents and adolescents understand SRH issues and how their personal experiences shaped this understanding. The second theme captures the constructions of intergenerational cultural norms and expectations that shape the lens through which individuals view SRH. How these norms differ and contribute to a collective framework that influences perceptions and behaviours related to SRH. Within this cultural framework, the third theme focuses on gender double standards, which emerge as a significant factor in participants conversations. The emphasis was on how societal expectations often create distinct norms for men and women regarding SRH. These double standards not only influence individual behaviours but also contribute to broader social dynamics and power structures. Amidst cultural norms and gender expectations, individuals acquire "streetwise" SRH knowledge, which forms the fourth theme. This informal and often experiential understanding of SRH is shaped by the realities of everyday life, providing a practical perspective that may deviate from formal cultural norms. Lastly, the fifth theme delved into how social media becomes a powerful influencer, amplifying and reshaping SRH narratives in the digital age. These themes depict the controversy in SRH communication within slum settings as described with excerpts below.

#### SRH meanings and experiences

The meaning attached to SRH by parents and adolescents, as well as their experiences, can vary and have the potential to shape the conversation around SRH issues. There is a consensus among the participants about the general meaning attached to SRH. Parents and adolescents were aware of the resultant effects of risky sexual behaviour such as HIV/AIDs, STIs/STDs, and unwanted pregnancy among others. The participants observed that protection against undesirable SRH outcomes requires self-discipline and resilience. However, the derivable pleasure in sexual activities could influence how individuals would position themselves against adverse SRH outcomes. With the information that the vignette character Adio got Abeni pregnant, the participants blamed and called both Adio and Abeni by different names. They were accused of negligence and failure to learn from other experiences. Parents gave anecdotal experiences of adolescents in their immediate community to substantiate the experiences of adverse SRH outcomes and how it affects adolescents. They acknowledged that such experiences were not uncommon in their community, given the unique characteristics of their environment. Parents affirmed that adolescents view early sexual encounters as a critical marker of maturity, which is why they are so prevalent. As such, they talk to their adolescents in one way or another because, according to them, such experiences have the potential to renew the cycle of poverty within the family and in the community at large. In parents’ words:

*What I see on this matter is that I have someone in my society that is not more than 11 years pregnant*. *People came to seek her at home*, *we told her parents that people coming to look for this girl at home is not good*, *and the parents will be saying this and that*, *until she was impregnated*. *When she was*, *it was the parents that took responsibility*. *Now*, *she is the one that is having her a child at the parents’ house* ……. *So*, *it is necessary to watch over children well*, *to be talk to them that all these behaviours are bad……* **(FGD/Men/45years/Ibadan Northeast**).

Narrating further experience, a 48year old woman said:

*The experience I have was when I was still in my hometown*, *there was a woman called Aunty Ronke*. *She was very loquacious; three men are not enough to meet her sexual desires for a day*. *Her parents always caution her that what she’s doing is wrong*, *but she always curses her parents*. *Eventually*, *she got pregnant*, *and they asked her who was responsible for the pregnancy*, *but she couldn’t hold anyone responsible out of all the guys she had sex with*. *She gave birth to the child and then she was given a room in her father’s house*. *After she gave birth*, *was she not supposed to be reasonable*? *But she was not reasonable enough and continued till she got her second pregnancy*. *Currently*, *she has three children in her father’s house*. *Whenever people passed by her house back then*, *they always made nasty comments about her*. *Whenever I go back home*, *she always cries when she sees me*. *Any child that they advise but won’t listen*, *it’s for the good of the child*. *It’s because of the future* **(FGD/Women/48years/Ibadan Southeast**)

Furthermore, when adolescents were asked if they understood SRH issues in general, the majority alluded that they understood what SRH issues are and their consequences. However, they admitted that they lack detailed knowledge of and proper guidance about SRH issues. Adolescent participants recounted some SRH experiences among their peers. They stated that adolescent girls are sometimes exploited sexually with the promise of financial gain. Many of the participants acknowledged that they have friends and neighbours who have had similar experiences in their neighbourhood. Some vulnerable places were highlighted in their communities where some adults, primarily men, sexually exploit and abuse young girls on a regular basis. It was argued that such a sexual encounter could be challenging to disclose to friends or their parents, and might pave the way for subsequent sexual engagement, whether voluntary or lured. Also, both boys and girls reported the experience of being set up for sexual intercourse, as well as having multiple sexual relationships. This suggests unequal power dynamics in their communities, which frequently make it more difficult for young girls to negotiate and navigate safer sex spaces. The particularity of their experiences is a common feature of adolescents living in slums as narrated below:

*I know a brother in this area who always plays with my friend*. *One day he bought ice cream and meat pie for her*, *took her to an uncompleted building and started touching her and doing all sorts of things with her*. *She tried to resist him; she struggled and cried*, *but he overpowered her*. *She did not tell anyone about what happened*, *but when she started having some discharge and itches in her private part*, *she spoke out to Aunty Temmy*, *who now suggested antibiotics to her*. *Up until now*, *the parents did not know what happened*. *One would think she has learned; she still plays with boys anyhow*, *and I even heard she is dating someone who is 7 years older than her*… **(FGD/Female Adolescent/18years/Ibadan Northeast)**

Throwing more light on the day-to-day experience in the community, a male adolescent reported that:

*It is very common in my community where I live*. *However*, *due to my strict parents*, *they do not allow me to associate with negative influences (bad people)*. *In our community*, *there is a specific group of individuals whose daily activities involve approaching and attempting to woo girls*. *I have witnessed numerous instances of unwanted pregnancies and unwanted children resulting from these interactions*. *This issue is unfortunately very common in my society*. **(FGD/Male Adolescent/18years/Ibadan Northeast)**

#### Intergenerational cultural norms and expectations of SRH

The majority of participants shared a collective understanding of cultural norms surrounding SRH issues. Parents believed in abstinence until marriage for adolescents, noting a shift from traditional values to contemporary times where early initiation into sexual activities is observed. Cultural norms and a perceived lack of contemporary knowledge often hindered parents from engaging in open conversations about SRH. Parents expressed concerns that such discussions might encourage experimentation which may lead to a tendency to provide simplistic advice or warnings against pre-marital sex. The generational gap further strained communication, as parents felt disconnected from their adolescents’ outlooks and cultural influences, resulting in a fundamental difference in perspectives on SRH issues. Excerpts from parents highlighted that adolescents’ dressing, the slang they use, and quickness to leave their parents’ house contribute to the challenges in bridging the intergenerational gap and facilitating effective communication on SRH matters. They also expressed concerns about the adverse effects of civilization on their community, noting that improved infrastructure and increased exposure to external influences have contributed to a decline in their cultural norms and values. The following quotes shed more light on parents’ perspectives:

*What we experience now was not common when we were still young*. *When we were young*, *we wore good clothes but nowadays*, *these children wear clothes that leave their body exposed*, *they use different slang*, *and they are quick to leave their parents’ custody*. *With the way females dress*, *even guys are tempted to stare at them*, *and this is the sole cause of what is happening nowadays because we expose what is not meant to be exposed*. *That is the major cause of everything we’ve been discussing*. *I would say*, *it is essential for parents to be proactive so that they can catch up with these children* **(FGD/Men/46years/Ibadan Southeast)**

In consonance with the above position, a community also expressed his view on cultural norms and expectations for SRH issues:

*Our civilization has brought harm to us*. *You will see some places are local and once a good road gets there*, *there is a problem*, *which is civilization*. *All their local acts will reduce*. *This civilization on watching all sorts is affecting them a lot and that is what they all like to watch* ……. *In the olden days*, *no one could have this talk*. *Even*, *you cannot say it then*, *there is shame and you cannot utter it but now if you wait a little while*, *you will see them come sit and they will be saying all sorts*, *you might have to block your ear when you hear things like–I have sexed her 6 times*, *he will not say sleep with*, *he will say sexed*. *That is how bad civilization has affected us*. *Parents must sit their children down and guide them appropriately*, *otherwise those children will go astray* **(KII/Community Leader/62years/Ibadan Northeast).**

Similarly, when adolescents were asked about cultural norms and expectations of SRH, they reported that their parents usually preach abstinence as culturally acceptable and expected behaviour for them. They further reiterated parents’ earlier position that choosing to remain a virgin until marriage is respectable behaviour and involves a personal conviction for both boys and girls. Some of the adolescents explained that abstinence from sex helps to protect adolescents’ SRH, preserve the sanctity of sex among adolescents, and keep the body clean. They submitted that their parents expect them to be good children and be of good conduct by not involving in any form of sexual or intimate relationship. The consensus was that early sexual initiation and premarital sex were bad and could result in undesirable consequences for them. Traditional measures of protecting oneself against undesirable consequences of sexual behaviour could be abstinence from sexual activities as well as proper parental guidance. However, many adolescents argued that this culture is eroding, and most adolescents hardly stay without having sex, as explained below:

…………. *Yoruba doesn’t give room for reckless living even though enlightenment allows it…*… *In the past*, *it was not so as we were taught as children would have been educated before time and would be prepared to be married off*. *So*, *our culture does not give room for premarital sex and unwanted pregnancy*. *So*, *I think parents will have to move with the current trend to be able to nurture their children well* **(FGD/Male Adolescent/18years/Ibadan Northeast)**

*Due to being the eldest daughter*, *my parents held specific expectations for my future*. *They consistently preached the importance of abstinence to avoid unplanned pregnancies*. *My mother*, *in particular*, *continually urged me to preserve my virginity and cautioned against allowing any man into my life at this moment*. *But*, *adhering to these expectations proves challenging*, *especially when faced with mockery from my friends* **(FGD/Female Adolescent/17years/Ibadan Southeast)**

#### Gender double standards in SRH

The discussion between parents and adolescents gave rise to gender double standards in sexual socialisation, which supports either masculinities or femininities. This category explored how discussions between parents and adolescents concerning SRH issues could serve yet another mechanism for the perpetuation of gender norms. Through gender double standards, parents unwittingly reinforce traditional gender norms which put pressure on adolescents to conform to preconceived notions about how adolescent girls and boys should act, behave, and interact based on their gender. Opinions are divided on this, as some parents’ favour preserving gender norms about sexuality as they are since doing otherwise would amount to teaching adolescents about having sex, which may be irresistible, while others believe gender norms need to change to meet their contemporary realities. Parents who believe they should retain the gender norm explained:

*In our culture*, *effort is made to train boys to behave like men and they have no problem about sexual issues because they will be the ones to marry their girls*. *In fact*, *boys have no problems except if they impregnate a girl or sometime contact gonorrhea and maybe other diseases that are now common*. *Contacting gonorrhea is a sign that they are sexually active*. *I think we should be more conscious of our girls because any little mistake on their part can jeopardise their ambition and will affect them greatly*. *I always encourage my wife to talk to our daughters and sometimes we warn our boy too* **(KII/Community Leader/62years/Ibadan Northeast).**

Some parents admitted that the world had changed and that parents needed to adapt to keep up with their children. It was submitted that regardless of their gender, parent must educate their children on sexual issues. They stated:

*I suppose* " *Ọmọ táráyé bí ni aráyé ń gbé p
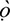
n" (the demands of time dictate how issues are addressed) is my way of saying that regardless of how we feel about it*, *parents must teach their children about sexual issues*. *If we don’t*, *they will find out about it in some way*. *Not even with how pervasive social media is*, *the information children receive from it*, *and those "agbaya"—nasty elders who have turned themselves into big sisters and brothers in this setting and do nothing but corrupt our children*. **(FGD/Men/49years/Ibadan Northeast)**

Another striking issue that came out in this conversation is the celebration and affirmation of masculinities among male parents. They argued that young men should exhibit their sexual skills to show young women that they are powerful. Conversations like this encourage young people to act in ways that conform to prescribed notions of masculinities and femininities. They also urge young men to engage in sexual activity while furthering the subjection of women. The discussion of SRH issues should focus on educating both male and female adolescents appropriately rather than upholding the traditional gender norm of male dominance. They modelled for their sons how to display their sexual prowess by speaking heroically about their own early sexual experiences. According to one of the parents:

*How can I have a son and be doing anyhow or sluggish with a girl*. *I usually tell one of my sons that he is not like me*. *Others are not like him*. *He is too sluggish*. *When I was their age*… *I was very sexually active*. *I like it when they report to them that they are following girls although they must face their studies*, *but it is one thing that shows they resemble their father*. **(FGD/Men/45years/Ibadan Northeast)**

#### Streetwise SRH knowledge

When asked where adolescents acquire their SRH knowledge from and how parents and adolescents feel about the effectiveness of parent-adolescent communication, there was consensus among parents and adolescents that most adolescents in their community acquire practical SRH knowledge on "the street," which indicates through their daily experiences in their communities. This SRH knowledge is often gained through sources, such as peers, aunties, family members, and the broader community, rather than through parents. Parents submitted that they often learn from their children when they hear them talk about certain issues regarding SRH. However, according to parents, the streetwise SRH knowledge may perpetuate harmful myths and misconceptions, such as the belief that certain sexual behaviours are markers of being sophisticated or civilised. There is the possibility that adolescents may also face pressure to conform to the dominant slum sexual culture, which can limit their ability to make informed decisions about their own sexual health. When reacting to the Adio and Abeni anecdote, some parents felt both adolescents are not streetwise, if they were, their parents would not have discovered Abeni was pregnant before they would abort it. They recounted that many adolescent girls have aborted in their community, and some women participants even affirmed that some mothers would have done the abortion before the father would know. As illustrated by one of the participants:

*Because they have a source for sexual information*, *these children of ours are more knowledgeable than we are*. *With children who already know more than what you want to discuss with them*, *how can you even begin a conversation on sexual matters*. *Children can be misled by happenings in our community because it is filled with atrocities*, *and the majority of the children pick up lessons from what happens in our neighbourhood*. *Some of us never had the opportunity to speak with their source*, *thus they have a different perspective on life*. **(KII/Women Leader/56years/Ibadan Southeast)**

Adolescents’ narrative also confirmed parents’ assertion that they obtain sexual information from the street. Adolescents explain that they are often curious and interested in learning about SRH topics such as puberty, sexuality, contraception, sexually transmitted infections (STIs), and abortion. They established that they would have loved to be educated by their parents, but their parents are judgmental most times. They affirmed, in many cases they turn to their peers, the internet, or other sources for information about SRH. While these sources can provide some helpful information, they can also be unreliable and may not provide accurate or comprehensive information. The participants adjudged that they feel comfortable discussing and learning about SRH issues among their peers. They argued that they can find any knowledge they need on the street, regardless of what it is. “They will update you on the situation” says one of the adolescents.

*“Ori street laye wa*”*–there is life on the street*. *If you are not there*, *they cannot inform you how things dey happen*. *What your parent can not tell you about sex*, *they will tell you oh ha on the street*. *Even if you fuck-up*, *people will tell you what to do*. *For instance*, *I contacted gono one time and it was one of my guys that told me what to take and I was fine*. **(FGD/Male Adolescent/19years/Ibadan Southeast)**

#### Social media exposure to SRH

Social media exposure shapes the context within which parent-child communication on SRH occurs. While acknowledging the high accessibility of SRH content through social media, study participants emphasized both positive and negative impacts of exposure to social media. Positive influences included communication and accessing health tips, while negative consequences involved exposure to explicit content and a lack of privacy. Parents participants expressed concerns that social media interference could distort the SRH education they provide, which may lead to potential confusion for adolescents. Despite the benefits, the overall sentiment was that the adverse effects of social media on SRH issues outweighed the positive impact, with participants linking media exposure to early sexual practices. They felt social media exposes adolescents to numerous unrestricted information that is damaging to their SRH while transcending the boundaries of parental authority. Parents recommended monitoring adolescents to guide them effectively in using social media for positive purposes. As articulated by the parents:

*It is useful for them although it does more harm than useful for children*. *At least for the purpose of communication so that they can be reached*, *if her mother is not near*, *so as to call her to meet her here or there*. *But these children are using it for different things*, *do you understand*? *You may see different things they view and watch on it*. *They now watch pornography*, *snap nude pictures*, *and send them to themselves*. *They use it to call themselves at midnight*, *chat on Facebook and so many other things*. *However*, *there are good reasons as some claim to buy it for their children for the sake of communication and to know their whereabout*. *I will only advise parents to monitor what their children are doing on it* **(FGD/Women/42years/Ibadan Southeast)**

Also, the community leaders emphasized the negative impact of social media exposure to SRH on adolescents and the potential consequences if left unchecked. They illustrated their point using a story narrated by a school principal involving an adolescent secretly recording her parents’ intimate moments. Their narrative underscores the importance of parental responsibility in guiding their children aright especially in situations where living conditions may contribute to adolescents experimenting adult behaviour. The following quote reflects their view:

*You see*, *the media have spoilt so many things*. *If unchecked*, *these children will wreak havoc*. *Parents have a major role to play*. *The reason why I said so is because there was a day I went to my child’s school for their P*.*T*.*A meeting and the principal told us a story of a family that lives*, *probably*, *in a single room apartment*. *The child is aware that the parents have sex every other night*, *so she carefully hid the phone in the wardrobe to record them*. *The principal said he was just passing by when he heard the chatters of some students that “your father will kill your mother”*, *so he stopped to see what they were doing*, *and he discovered that they were all girls*. *When he requested the phone*, *they switched it off*. *He then put it on and asked them to show him the particular video they were chattering about*. *When she eventually showed him*, *he invited her parents to school the following morning*. *When the parents came the following morning*, *the mother was ranting that the phone belonged to her child*, *that she bought the phone for her*. *The principal showed her the video*, *then she became silent and left the principal’s office in shame*. *And that is why I said parents have the major part to play*, *since they know they live in a single room apartment and the child is all grown up but still sleeps in their room*, *and it is impossible for couples not to have sex*. *They may think the child is asleep*, *meanwhile he is not*. *So*, *the child will also want to practice such outside*, *since he knows his parents enjoy it*. *The parents have a major role to play in issues like this* **(KII/Community Leader/60years/Ibadan Southeast).**

Adolescents expressed similar views with that of parents about media exposure to SRH. The majority also felt that media have both positive and negative impact on adolescents as earlier stated by parents. Adolescents acknowledged that watching sexual content on media devices could predispose them to early sexual activities. Many adolescents argue that they learn so many things on social media, especially things that their parents do not have knowledge of or could not explain to them. They believed social media is a good thing for them, however, parents should train their children well to differentiate what is good or bad on social media. The excerpt below is reflective of their position:

*Social media has contributed both positively and negatively to our development*. *Positively in the sense that*, *there are some health issues that they make us know and negatively in the sense like watching of porn videos*. *Because when a girl or boy gets to watch porn video*, *he will want to put it into action because he has watched the demo*, *he too wants to get the real one to himself*, *but parents can checkmate their children exposure to such things by occasionally check through their phones or guide what they watch on the TV* **(FGD/Male Adolescent/16years/Ibadan Northeast)**

Adolescents’ sexual activities are shrouded in secrecy because they tend to comply in public by lying about their sexual behaviours and presenting themselves as well-behaved, especially to their parents. When relating their conversation about social media exposure to SRH discussion between parents and adolescents, some adolescents believe their parents are unaware of the current trends regarding their SRH issues. Many adolescents are curious about the sexuality depicted in popular culture and online. Adolescents stated that their parents are not "tech-smart" enough to know what they are doing on social media. Female adolescents claimed that they could act in a way that would satisfy their parents, such as staying indoors and acting responsibly, since their parents want to keep an eye on their whereabouts. Nonetheless, they admitted that when it comes to SRH behaviour, they cling to the trends, such as picking up slang from celebrities and attempting to act acceptably around their peers. They acknowledged that they could get their hands on sex toys and objects like dildo while their use of social media platforms fuels their desire. Many adolescent males, however, reported that some of their peers indulged in masturbation. It was asserted that using social media to meet, communicate with, and conduct sexting or video sex with partners has made practicing SRH behaviours easier. Adolescents of both sexes agreed that they used one or more social media sites, and that they enjoyed doing so because it was entertaining. One of them narrated:

*I laugh when my mummy asks me questions like*, *Are you sexually active*? *Well*, *in my mind*, *I am*, *but she must not know*. *How will she know when I’m supposed to be strongly guided by her*? *So*, *the answer is no*, *I’m not*. *Meanwhile*, *I get my pleasure and satisfaction from my vibrator (sex toy)*. *Some of my friends do it too*…… *Have you ever heard the word "happy pills"*? *Well*, *it is a pill we put in drinks to get very horny and active while using the toys and yet be the good girls to mummy…*

(FGD/Female Adolescent/19years/Ibadan Northeast)

Another adolescent offered the following from his own experience:

*My daddy thought he knew how to use the phone; he always complains that I spend too much time with my phone*. *He then seized my phone to see what I always do*, *he searched and searched and couldn’t find anything*. *Me that I have my hidden app*, *I hide everything inside app lock*. *He returned the phone to me… as for porn*, *boys have porn on their phone but hidden*…. *The truth is except if you people want to deceive yourself*, *you are at one time watch porn or do video sex …so stop pretending*.

(FGD/Male Adolescent/19years/Ibadan Southeast)

## Discussion of findings

The study aimed to provide qualitative evidence on the situational context of parents and adolescents’ communication about SRH issues in the urban slums of Ibadan, Nigeria. The study found that events in the slum environment significantly influence parent-adolescent discussion about sexual issues, which further undermines and distorts the normative roles of parents in informal sexual education. This is reflected in the five categories that emerged from the FGD and KII participants’ discussions. The study revealed that while sexual issues like risky sexual behaviours and their negative consequences were widespread in their communities, parents and adolescents shared common understandings of them and valued SRH conversations. This result is similar to results of other studies conducted in Ghana [52, 53], Ethiopia [19, 54], Nigeria [21], and SSA [55]. However, the findings contrasted with the study who found low level of parental understanding of SRH issues and their consequences in Nepal [56]. As argued by social constructivism, the emphasis on shared meaning transcends just a shared understanding of SRH issues and incorporates a process of negotiation, interpretation, and mutual agreement between parents and adolescents on SRH issues. Thus, it can be inferred that the rationale for the ineffectiveness of parent-adolescent communication about SRH issues is the inability of parents and adolescents to negotiate, interpret, and reach mutual agreements on how best to communicate and what to communicate about in terms of SRH issues.

Participants viewed sexual activity as a dynamic social experience that results from a dialectical relationship between them and their social environment. Parents and adolescents recounted various anecdotal experiences of sexual exposure, rape, exploitation, sexual violence, and adverse health outcome in their communities. Similar findings about exposure to risky sexual behaviours among adolescents in Africa, including early sexual debut, involvement in multiple sexual partnerships, and condomless sex, were reported by [57] and [58]. Previous research has demonstrated that sexual violence, such as rape, has exposes adolescent girls and women to HIV/AIDS and other STDs, as well as the likelihood of being impregnated by their perpetrator and having an HIV-positive foetus [59]. In addition to sexual violence, parents and adolescents acknowledged that their community paved the way for sexual exploitation of adolescents which sometimes makes parent-adolescent communication ineffective. Evidence from a study in SSA showed that sexual exploitation in the form of transactional sex enhanced adolescent boys’ and girls’ chances of having multiple sexual partners [60]. This indicates that the continuous exposure and engagement in risky sexual behaviours by adolescents creates an avenue for parents, who are the key sources of information, to educate their adolescents on SRH issues. Unfortunately, despite parents’ efforts to ensure their children receive quality SRH education, it appears that the SRH information they are providing does not correspond to their children’s reality or to events in their neighbourhood.

Further, the dynamics of cultural norms and expectations of SRH in society resulted in different worldviews for parents and adolescents about SRH issues. Both parents and adolescents affirmed that cultural norms and expectations around sexuality are changing. The finding showed that parents were strongly attached to traditional norms of abstinence and the expectation that adolescents should be conservative in their approach. On the contrary, adolescents reported attachment to a new reality of changing sexual experimentation and exploration, although they highlighted that parents expected them to abstain. The study observed that the intergenerational differences between parents and adolescents’ cultures had influenced SRH communication. This diverse worldview between parents and adolescents may have accounted for unstable SRH discussion or the adoption of an indirect communication pattern earlier reported by previous studies [27, 28, 61, 62]. This approach stemmed from the fact that parents are sometimes strict, may not have adequate knowledge of SRH issues and/or may not be comfortable discussing with their adolescents [21, 63, 64]. This may have undermined the need for open and responsive parent-adolescent communication about SRH issues [65]. This finding aligns with previous findings that traditional norms or cultural practices inhibit and create a communication gap between parents and their adolescents on SRH issues [31, 38, 66, 67]. Also, it is consistent with [13] findings that adolescents with negative behavioural beliefs and subjective norms about discussing sexual matters with parents, and having a perception of their parents having poor SRH knowledge, were more inclined to engage in ineffective SRH communication. Drawing from social constructivism, the theory elucidates the impact of power dynamics on shaping cultural norms and expectations regarding SRH. Dominant cultural groups wield more influence, potentially sidelining marginalised groups in constructing these norms. This is true for parents and adolescents in SRH conversations. While adolescents, who are a marginal group, sought a dialogue that reflected their genuine SRH situation, parents, who are the dominant group, want to enforce conservativeness; as a result, conversations about SRH are focused on sexual chastity and abstinence.

Moreover, gender double standards in SRH revealed that adolescent boys and girls receive informal sexual education in ways that are different from one another and perceived to be in accordance with established gender norms. The study found a two dimension to unequal gender relation in the communication of SRH issues. In their discussions of SRH, parents frequently give less attention to adolescent boys. Typically, the conversation is passive for male adolescents. However, adolescent girls are given a lot of attention, presumably because they are the most affected by or at risk for negative SRH consequences. Meanwhile, a study has earlier explained that girls are more likely than boys to get home-based sexuality education, in large part due to the widespread construction of adolescent girls’ sexuality as dangerous and vulnerable [68]. On the other hand, parents encourage males to pursue girls in their messages, promoting masculinities, while encouraging girls to practise conservative sexual behaviours, promoting femininities. This gender double standard influences which gender of parent converses with their children more and makes parent-adolescent conversations about SRH issues less effective. From this finding, two dimensions can be inferred. One is that the gender double standard might influence how comfortable parents are and how willing adolescents are to talk about sexual issues. This strengthens the contention that parents and adolescents may feel more comfortable discussing sexual topics with the same gender, especially when discussing sensitive topics such as menstruation, sexual orientation, or gender identity. Evidence from earlier studies shows that fathers discuss SRH issues with boys, whereas mothers converse with girls more about SRH issues [38, 69, 70]. In contrast, [70] found opposite gender communication on SRH between parents and adolescents. Second, adolescent girls receive greater attention than boys when discussing sexual health issues, which shows that mothers are the primary educators of their children’s sexuality while fathers played passive role. [32, 36], and [71] reported a similar finding that due to the perception that daughters are more vulnerable than sons, most mothers preferred to have this conversation with their daughters, although very few mothers claimed they would feel more at ease talking about it with their sons. The discourse with adolescent boys is constrained since they are less likely than females vulnerable to SRH consequences [3, 36, 72]. It can be inferred that this nature of communication left out adolescent boys, as important SRH issues were not consciously discussed with them. Thus, gender double standards in SRH discussion limit parents’ ability to communicate freely with their children and potentially lead to unbalanced or incomplete information being shared. Further studies are required to explore gender inequality in SRH discussions between parents and adolescents.

The study also showed that streetwise SRH knowledge distorts parent-adolescent conversation about SRH issues. It is glaring in the FGDs conversations that adolescent’s access and acquire SRH information from their peers, some area brothers and aunties, and others in the community who all support the same SRH experiences and behaviours. Most of the information they learn on the street contrasts sharply with what they discuss with their parents. This finding resonates partly with the findings of [70, 72], and [73] who claimed that paternal aunts or uncles are primary source of SRH information for adolescents. Also, the finding is consistent with the submission of [74] who found that adolescents are now turning to their peers and social media for SRH education as a result of the decline in extended family relationships. The findings of the present study, however, go beyond peers, aunts, and uncles to examine community events that serve as streetwise SRH information and their role in distorting SRH conversations between parents and adolescents. Consequently, the finding implies that if the conversation does not include any potential street-smart SRH knowledge that adolescents may acquire, the SRH discussions between parents and adolescents will not be productive.

Furthermore, social media exposure to SRH was found to interfere with the parent-adolescent discussion of SRH issues. Exposure to social media played an influential role in adolescents’ source of SRH information. The finding illustrated that adolescents are immersed in uncensored SRH information and contents on social media, hence may prefer sourcing SRH information from the media. Both parents and adolescents observed that exposure to sexual content on social media is connected to early sexual practices among adolescents. This finding is in consonance with the findings of [73, 75] and [76]. These studies demonstrated that watching sexual content in the media has a negative impact on the sexual activity of adolescents. According to [75], media contents regarding sexuality may be particularly tempting to adolescents because media users are more prone to adopt behaviours portrayed by characters that are viewed as attractive and realistic.

Conversely, some studies have established the positive effect of exposure to social media on adolescents’ sexual behaviours. For instance, a study by [77] found no correlation between young peoples’ exposure to sexual content on television and their participation in sexual activity. Also, [78] study supported this finding. In their study, [78] demonstrated that sexy media has no correlation to either sexual initiation among youth or sexual behaviours. In line with these findings, [79] affirmed the positive impact of social media on adolescents’ sexual behaviours. In all, these authors argued that social media could serve as a powerful tool for sexual education as well as if proactively used it will increase parent-child communication about SRH issues. One possible explanation for the variation in the study’s findings could be that young people have seen social media as a source of information to improve their sexual behaviour. The role of social media in general poses a significant challenge to parent-adolescent SRH discussions in SSA.

Overall, the findings of this study gave credence to [46] postulate that the community context shapes peoples’ attitudes, beliefs, and perceptions as they interact within society. This is evident in how parents and adolescents construct their day-to-day SRH reality. Within this framework, there exists a collective understanding of responsible SRH behaviours and otherwise. Both parents and adolescents recognise the prevalence of negative SRH behaviours in slums, influenced by the challenging conditions in these contexts. As a result, the interpretation of SRH realities and experiences by parents and adolescents influences discussions about SRH issues, shaped by their individual perspectives and experiences. Intergenerational norms and expectations hinder communication about SRH, as adolescents may perceive their parents as old-fashioned, while parents may see adolescents as stubborn and unreceptive to learning about SRH issues. The differences in constructing their respective realities impede conversations and learning about SRH issues. Gender double standard shows that the construction of boys’ and girls’ realities and SRH socialization differs. The patriarchal nature of the slum context encourages boys’ prowess in SRH, while girls are most often socialized to conform to gender norms. The reality of streetwise SRH knowledge indicates that adolescents in slums often construct, code, and decode SRH knowledge on the street. Parents may lack awareness of the codes and slangs used by adolescents, hindering their understanding of their realities. Social media exposure is another emerging reality parents grapple with in providing adequate and responsible SRH knowledge to adolescents. Meanwhile, studies consistently emphasize that parents are in the best position to educate their adolescents on SRH issues [5, 54, 56]. Therefore, in constructing SRH narratives, situations around adolescents should be considered to ensure the dispensation of accurate and relevant SRH information.

## Strengths and limitations

Due to scant evidence from previous research on situational context, the study employed a vignette-based qualitative approach to delve into the situational context of SRH communication between parents and adolescents urban slum dwellers (a conservative setting). However, it is important to note both the strengths and the limitations of this study. In terms of strengths, the use of vignette-based FGD and KII mixes provides robust and complementary data from the more in-depth perspective of parents, adolescents, and community leaders. The recruitment of the participants was done by the gatekeepers, who were trusted and respected by the community, which helped parents and adolescents feel more comfortable discussing SRH issues. Also, the study employed social constructivism as its foundation and applied its principles to explain how parents and adolescents construct and perceive SRH reality. Despite the highlighted strengths, this study has some limitations. Although vignette-based FGD allowed for deeper discussion and conversation regarding SRH issues, those with quieter voices or minority opinions found it difficult to express themselves. This was mitigated by consciously seeking their opinion on the issues being discussed. In addition, participants discussed the vignette stories and responded to questions based on the vignette characters rather than directly discussing issues of SRH communication in their own families. This may have limited the validity of the information provided. However, the prefield visit anticipated this, and the anecdotal experiences in the vignette illustrate the daily realities of their surroundings, which help to minimise it. Finally, the quality of the SRH information that participants in this study received from the influential members of their community was not examined, and it is unclear how this would support informal sex education. Future research might want to explore this.

## Conclusion

This study found that situational context such as SRH meanings and experiences, cultural norms and expectations for SRH, gender double standards, streetwise SRH knowledge, and social media exposure to SRH inhibit effective parent-child communication SRH issues. Inference from parents’ and adolescents’ anecdotal experiences indicates that parents must educate their adolescents about sexuality in a context-appropriate manner (sexual knowledge that will aid them in navigating their surroundings) without downplaying the fact that adolescents are already sexually active. To achieve a balance between the sexual worldviews and reality of parents and adolescents, interventions must be specifically designed to consider the popular culture of adolescents. Such interventions should provide cultural synthesis of conservative and current sexual norms and consider neighbourhood actors who expose adolescents to sexual behaviours or exploration. The interventions must factor-in the importance of streetwise SRH information and social media as sources of sexual socialisation for adolescents. Ultimately, promoting SRH among adolescents in slums requires a comprehensive and collaborative approach that considers the unique challenges and contexts of these communities. This may involve working with community leaders, parents, and adolescents themselves to address gender double standards and cultural norms around sexuality, as well as creating safe spaces where adolescents can access confidential sexual health services without fear of judgement or discrimination.
